# Determinants of maternal healthcare service utilisation among Indonesian mothers: A population-based study

**DOI:** 10.12688/f1000research.73847.1

**Published:** 2021-11-08

**Authors:** Ridwan Setyo Aji, Ferry Efendi, Iqlima Dwi Kurnia, Santo Imanuel Tonapa, Chong-Mei Chan

**Affiliations:** 1 Faculty of Nursing, Universitas Airlangga, Surabaya, Indonesia; 2 Research Group of Community Health, Surabaya, Indonesia; 3 School of Nursing, Faculty of Medicine Universitas Sam Ratulangi, Manado, Indonesia; 4 Department of Nursing Science, Faculty of Medicine University of Malaya, Kuala Lumpur, Malaysia

**Keywords:** antenatal care, institutional deliveries, maternal health, postnatal care, pregnancy

## Abstract

**Background: **In Indonesia, maternal health care services are widely available, aiming to improve health and survival among mothers. However, these services remain underutilised, and its determining factor was unknown. This study sought to identify determinant factors of maternal healthcare services utilisation among Indonesian mothers.

**Methods: **This population-based cross-sectional study leveraged the 2017 Indonesia Demographic and Health Survey data. A total of 12,033 mothers aged from 15 to 49 years who had a live birth in the five years preceding the survey were included in the analysis. Multivariable logistic regressions were used to identify the determinant factors.

**Results:** Approximately 93.44% of the mothers had adequate antenatal care, 83.73% had a delivery at the healthcare facility, and 71.46% received postnatal care. The mother’s age and household wealth index were the typical determinants of all maternal healthcare services. Determinants of antenatal care visits were husband’s occupational status, the number of children, and access to the healthcare facility. Next, factors that drive mothers’ delivery at the healthcare facility were the mother’s education level, husband’s educational level, and residential area. The use of postnatal care was determined by the mother’s occupational status, husband’s educational level, number of children, wealth index, access to the healthcare facility, and residential area.

**Conclusions:** Although there were differences in the determinant factors of three key maternal healthcare services, the mother’s age and household wealth index were the typical determinants of all maternal healthcare services utilisation. Providing a tailored programme aligned with these determinant factors may ensure that mothers can access and adequately utilise maternal healthcare services.

## Introduction

Maternal mortality is a serious global health problem that threatens healthcare systems. In 2017, approximately 810 mothers died every day from preventable causes related to pregnancy and delivery around the globe. ^1^ A vast majority of these mortalities occurred in low and middle-income countries, of which Indonesia is one. ^2^^,^^3^ In Southeast Asia, the maternal mortality rate among mothers aged 15-49 reached 16,000 per 295,000 mothers death or 5.4% of overall maternal mortality worldwide. ^1^ In Indonesia, the maternal mortality rate was about 305 deaths per 100,000 live births in 2015. This number was still threefold higher than the Millennium Development Goals (MDGs) target set up previously. ^4^^,^^5^ The Indonesian government’s challenge will be more demanding as United Nations (UN) shifted to the new goals. The 2030 Sustainable Development Goals (SDGs) launched by the UN in 2016 was aimed to decrease maternal mortality to 70 deaths per 100,000 live births. ^4^ Thus, reaching SDG’s target will be a considerable challenge for Indonesia.

The utilisation of maternal healthcare services (MHC) among mothers remains a challenge for the health system across the countries. Adequate utilisation of MHC is crucial to reducing maternal mortality and improve pregnant mothers’ well-being. However, maternal healthcare service utilisation among developing countries was relatively inadequate. ^6^^–^^8^ Particularly in Indonesia, it was reported that Antenatal care (ANC) visits in 2017 only reached 70%, which was lower than the government’s target of 76%. ^9^ Mothers in favour of Intra-natal care (INC) at the healthcare facility was about 62%, which was below the targeted rate by the government at 85%. ^10^ Moreover, only 48% of mothers used the Postnatal care (PNC) service, where is still far below the expected target at 80%. ^11^ Since the maternal healthcare service has been underutilised among Indonesian mothers and it is much lower than the government target, hence an understanding of its determinant is deserved further investigation.

Studies across the countries have built knowledge for associated factors with the utilisation of MHC. For example, previous works by Kurniati *et al.* had identified that MHC utilisation among mothers influenced by education, occupation, age at first delivery, contraceptive use, age at first marriage, participation in household decision-making, and attitudes towards intimate partner violence. ^12^ However, a previous systematic review of studies in the lower and middle-income countries underscores that determinants for maternal healthcare service varied greatly in each population. ^13^ Furthermore, numerous studies reported that determinants in three key components of MHC namely ANC visit, institutionalised delivery, and PNC use were considerably different. ^9^^,^^10^^,^^12^ Nonetheless, none of the studies carried out previously provide the entire picture about the determinants of these three keys to MHC utilisation. Based on these gaps, a study with representative data generated from the population level is deemed necessary.

Investigating determinants of MHC utilisation among Indonesian mothers requires nationally representative data because it can control ethnic variability among the participants. Drawing from the 2017 Indonesian Demographic Health Survey (IDHS), this population-based study sought to identify determinant factors of MHC utilisation (ANC visit, institutionalised delivery, and PNC use) among Indonesian mothers. An understanding of determinants in each service could provide evidence for the government to develop a personalised intervention for Indonesian mothers based on the type of services.

## Methods

### Study design

This population-based, cross-sectional study used nationally representative data from the IDHS in 2017. This survey is part of the International Demographic and Health Survey (DHS) program, and its details can be found on the DHS program website (see the data availability statement for more detail).

### Study setting and population

The population in this study was households from 34 provinces in Indonesia. The IDHS collected data from July 24 ^th^ to September 30 ^th^, 2017, using a two-stage stratified sampling method. The first stage was selecting census blocks based on the wealth index that resulted in 1,970 blocks. In the second stage, 25 ordinary households were chosen from each block with systematic sampling from the list. A total of 49,627 women of childbearing age met the 2017 IDHS criteria to be interviewed. In the present study, the inclusion criteria of respondents included mothers aged 15 to 49 years who had a live birth in the five years preceding the survey. The exclusion criteria were whether the variables incomplete or missing. Of these, a sample of 12,033 mothers from 34 provinces in Indonesia was analysed.

### Variables

The outcome variable in this study comprised the three key MHC components, namely ANC visits, institutionalised INC, and PNC after delivery obtained from participants self-reportedly. The ANC visit was categorised under two levels: adequate (4 times or more) and inadequate (fewer than 4 times). Institutionalised INC defined as a delivery that occurred at a healthcare facility regardless of its types (clinic, public health centre, and hospital) and ownership (government-owned and private sector). The use of PNC refers to a health check for the mother after delivery. According to the Ministry of Health Republic of Indonesia, PNC has to be carried out at least three times within the first six hours to the third day after delivery, on the fourth day to 28 ^th^ after delivery, and the 29 ^th^ day to the 42 ^nd^ day after childbirth.

The explanatory variable in this study consisted of three categories that include mother’s sociodemographic factors, husband’s sociodemographic factors, and household-related factors. Under the category of mother’s sociodemographic factors, there were three variables, namely mother’s age in years (19-24, 25-34, and 35-49), mother’s occupational status (employed and not employed), mother’s educational level (primary, secondary, and higher). Three variables were under the category of husband’s sociodemographic factors consisted of an age gap with husband (younger/older than their husband), husband’s occupational status (employed and not employed), husband’s educational level (primary, secondary, and higher). In regard to household-related factors, in this category, there were three variables comprised the number of children (0, 1-3, and more than 3), household wealth index (richest, rich, middle, poor, and poorest), access to a healthcare facility (problem and not), and residential area (urban and rural).

### Statistical analysis

Data obtained from the DHS dataset were analysed using STATA version 16.0 (Stata Corp, College Station, TX, USA). Data were analysed using the descriptive statistics method, and results were presented as weighted frequencies and percentages. The adjusted odds ratios (OR; AOR) with 95% confidence interval (CI) of factors associated with MHC (ANC, INC, and PNC) were estimated by multivariate logistic regressions.

### Ethical consideration

The DHS program provided approval to use the 2017 IDHS data, and the data set is publicly available on DHS’s website. The 2017 IDHS study protocol has been approved for ethical clearance from the national board review of the Ministry of Health of Republic Indonesia and Inner City Fund (ICF) Macro institutional review board (number 45 CFR 46). Before participants were interviewed, informed consent was sought by each interviewer.

## Results

### Population characteristics

A total of 12,033 mothers were eligible for data analyses in this study. Table 1 presents the descriptive characteristics of the study population. A high proportion of mothers were aged from 25 to 34 years (55.26%), employed (50.08%), had secondary education (61.36%), and were predominantly younger than their husbands (81.45%). The majority of husbands were working (99.48%) and had received their secondary education (60.19%). In this study, the mothers had predominantly had 1 to 3 children (95.73%) in their household, considered themselves as rich (21.68%), mostly did not have a problem (89.83%) when accessing health facilities, and resided in the rural area (50.44%).

**Table 1.** Participants characteristics (n = 12,303).

Variable	n	%
**Mother’s age (years)**		
19-24	2,584	21.47
25-34	6,649	55.26
35-49	2,800	23.27
**Mother’s occupational status**		
Employed	6,026	50.08
Not employed	6,007	49.92
**Mother’s educational level**		
Primary	2,743	22.80
Secondary	7,304	61.36
Higher	1,906	15.84
**Age gap between mother and husband**		
Younger than husband	9,801	81.45
Older than husband	2,232	18.55
**Husband’s occupational status**		
Employed	11,970	99.48
Not employed	63	0.52
**Husband’s educational level**		
Primary	3,108	25.82
Secondary	7,242	60.19
Higher	1,683	13.99
**Number of children**		
0	480	3.99
1-3	11,522	95.73
>3	33	0.28
**Household wealth index**		
Poorest	1,032	16.88
Poor	2,433	20.15
Middle	2,587	21.50
Rich	2,609	21.68
Richest	2,302	19.79
**Access to healthcare facility**		
No problem	10,819	89.83
Problem	1,224	10.17
**Residential area**		
Rural	6,069	50.44
Urban	5,964	49.56

### The proportion of maternal healthcare services utilization among Indonesian mothers

**Figure 1.**
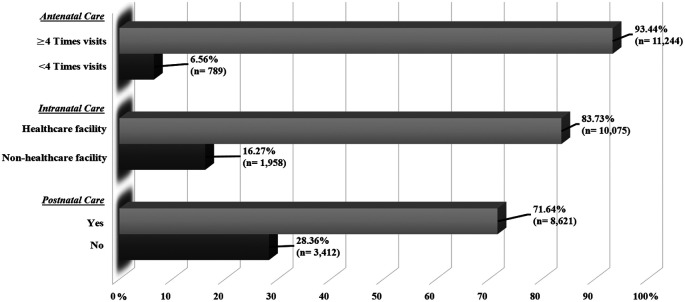
The proportion of maternal healthcare services utilization.

Figure 1 displays the proportion of Indonesian mothers’ maternal healthcare service utilisation in ANC, INC, and PNC. About 93.44% of mothers had adequate ANC visits (≥4 visits), while only 6.56% had inadequate ANC visits (<4 visits). Regarding institutionalised INC, 83.73% of mothers had delivered at the healthcare facility, whereas about 16.27% did not receive the delivery at the healthcare facility. In terms of the use of PNC, only 71.64% of mothers had the PNC after delivery.

### Factors associated with maternal healthcare services utilization

The multivariate logistic regressions analysis revealed five determinant variables of ANC visits ( Table 2). A higher odds of adequate ANC visits was found for mothers aged 35-49 years (AOR = 2.14; 95%CI = 1.55–2.94) and those aged 25-34 years (AOR = 1.48; 95%CI = 1.19–1.84) compared to mothers aged 15-24 years. Mothers who had employed husbands had a 2.91 (95%CI = 1.39–6.09) increased likelihood of having adequate ANC visits than those who were not working. Mothers with 1 to 3 children had 1.66 times (95%CI = 1.12–2.4) greater in having adequate ANC visits than mothers who do not have children. Compared to mothers who were in the poorest category, those who were in richest, rich, middle, and poor had 5.93 (95%CI= 3.81–9.20), 3.04 (95%CI= 2.18–4.25), 2.07 (95%CI = 1.56–2.75), and 1.87 (95%CI = 1.44–2.42) times likely to have adequate ANC visits respectively. Mothers who considered that access to the healthcare facility was not a problem had a 1.55 greater odds (95%CI = 1.20–2.02) of having adequate ANC visits than those who had problem accessing the healthcare facility.

**Table 2.** Factors associated with maternal healthcare services utilization.

Variables	Antenatal care			Intranatal care			Postnatal care		
	AOR	95% CI		AOR	95% CI		AOR	95% CI	
		Lower	Upper		Lower	Upper		Lower	Upper
***Mother’s socio-demographic factors***									
**Mother’s age (Years)**									
15-24	*Ref*			*Ref*			*Ref*		
25-34	1.48 *	1.19	1.84	1.19 *	1.01	1.41	1.23 **	1.06	1.43
35-49	2.14 ***	1.55	2.94	1.92 ***	1.52	2.43	1.31 ***	1.09	1.58
**Mother’s occupational status**									
Not employed							*Ref*		
Employed							1.15 **	1.03	1.27
**Mother’s educational level**									
Primary				*Ref*					
Secondary				1.57 ***	1.33	1.85			
Higher									
***Husband’s socio-demographic factors***									
**Age gap between mother and husband**									
Older than husband									
Younger than husband									
**Husband’s occupational status**									
Not employed	*Ref*								
Employed	2.91 **	1.39	6.09						
**Husband’s educational level**									
Primary				*Ref*			*Ref*		
Secondary				1.20 **	1.02	1.40	1.20 **	1.05	1.37
Higher									
***Household-related factors***									
**Number of children**									
0	*Ref*						*Ref*		
1-3	1.66 *	1.12	2.40				1.64 ***	1.31	2.05
> 3									
**Household wealth index**									
Poorest	*Ref*			*Ref*			*Ref*		
Poor	1.87 ***	1.44	2.42	2.14 ***	1.79	2.56			
Middle	2.07 ***	1.56	2.75	2.57 ***	2.07	3.20	1.30 **	1.08	1.55
Rich	3.04 ***	2.18	4.25	3.44 ***	2.71	4.37	1.29 *	1.07	1.56
Richest	5.93 ***	3.81	9.20	9.22 ***	6.54	13.00			
**Access to a healthcare facility**									
Problem	*Ref*						*Ref*		
No problem	1.55 **	1.20	2.02				1.23 *	1.03	1.46
**Residential area**									
Rural				*Ref*			*Ref*		
Urban				2.44 ***	1.96	3.03	0.81 **	0.70	0.90

*Note*: AOR: Adjusted odds ratio; CI: Confidence interval.

* : p < 0.05.

** : p < 0.01.

*** : p < 0.001.

Regarding institutionalised INC, five determinant variables were identified as statistically significant ( Table 2). A higher odds of delivering at a healthcare facility was found for mothers who aged 35-49 years (AOR = 1.92; 95%CI = 1.52–2.43) and aged 25-34 years (AOR = 1.19; 95%CI = 1.01–1.41) compared to mothers aged 15-24 years. Mothers who had secondary education had 1.57 times higher chances of delivering at healthcare facilities (95%CI = 1.33–1.85) than those with primary education. Also, mothers who had husbands with secondary education had 1.20 times more chances to deliver at healthcare facilities (95%CI= 1.02–1.40) than those with primary education. Compared to mothers who were in the poorest category, those who were in richest, rich, middle, and poor were 9.22 (95%CI = 6.54–13.00), 3.44 (95%CI = 2.71 4.37), 2.57 (95%CI = 2.07–3.20), and 2.14 (95%CI = 1.79–2.56) times likely to deliver at a healthcare facility respectively. Moreover, mothers who resided in urban areas had a 2.44 greater odds (95%CI = 1.96–3.03) of delivering at a healthcare facility than those living in the rural area.

With regard to the use of PNC, seven determinant variables were identified as statistically significant ( Table 2). A higher odds of PNC utilisation was found for mothers aged 35-49 years (AOR = 1.31; 95%CI = 1.09–1.58) and aged 25-34 years (AOR = 1.23; 95%CI = 1.06–1.43) compared to mothers aged 15-24 years. Mothers who were employed had 1.15 (95%CI = 1.03–1.27) times increased likelihood of utilising PNC after delivery than those who were not employed. Mothers who had husbands with secondary education had 1.20 times more chances to utilise PNC (95%CI = 1.05–1.37) than those with primary education. For mothers who had 1 to 3 children had 1.64 times (95%CI = 1.31–2.05) to use PNC than mothers who do not have children. Compared to mothers in the poorest category, those in the middle and rich categories were 1.30 (95%CI = 1.08–1.55) and 1.29 (95%CI = 1.07–1.56) times likely to use PNC. Also, mothers who considered that access to the healthcare facility was not a problem had a 1.23 greater odds (95%CI = 1.03–1.46) utilise PNC than those who thought that access to the healthcare facility had a problem. Moreover, mothers who resided in urban areas had 0.81 times (95%CI = 0.70–0.90) less chances of having PNC after delivery than those in rural areas.

## Discussion

The present study sought to identify determinant factors of MHC utilisation (ANC visit, institutionalised delivery, and PNC use) among Indonesian mothers population using the 2017 IDHS data sets. The results demonstrated that MHC utilisation among Indonesian mothers were relatively high. The main findings from the present study indicated that there were commonalities and differences in the determinant factors of three key MHC, which echoes findings from other studies in the lower and middle-income countries. ^13^^–^^15^ Among Indonesian mothers, age and household wealth index were the typical determinants of MHC utilisation. Concerning the ANC visits, husband’s occupational status, the number of children, and access to the healthcare facility were identified as its specific determinants. Next, factors that drove mother’s delivery at the healthcare facility (INC) were the mother’s education level, husband’s educational level, and residential area. In addition, the use of PNC determined by several factors includes the mother’s occupational status, husband’s educational level, access to the healthcare facility, number of children, and residential area. Based on this finding, healthcare professionals should be considering these determinants in providing MHC to Indonesian mothers. Furthermore, a wide-system effort is required from the government sector to develop a programme tailored with these determinant factors that can ensure MHC can be accessed and adequately utilised by mothers.

Age was an essential determinant factor for utilising ANC, institutional delivery, and PNC use among Indonesian mothers. Older mothers tend to utilised MHC correctly than younger mothers. This was thought because older mothers are more mature in appraising the benefit of using MHC. As older mothers are more experienced in pregnancy, they consider ANC service necessary during pregnancy, delivery at a healthcare facility relative safe, and received PNC will be essential for mothers and their babies. ^16^^,^^17^ This finding is definitely important because Kurniati *et al.* found that about 32% of mothers give birth for the first time when they were younger than 19 years old. ^12^ Therefore, developing effective policies for improving MHC among younger mothers is deemed necessary.

The household wealth index was identified to play a crucial role in determining utilisation of ANC visits, institutionalised delivery, and the use of PNC. Compared to mothers from the lower bound of wealth index, those in high wealth index were prone to have adequate ANC visits, delivery at the healthcare facility, and utilised PNC services, which was in line with findings from the previous study. ^12^^,^^14^ The wealth index was well known as a structural factor affecting mothers’ ability to seek healthcare services through multiple mechanisms, including the ability to commute, financial capacity, geographic accessibility, and ability to comprehend the context of such services. ^18^ In doing so, those who are living in poverty condition are at risk for underutilised maternal health services. This finding warrants attention because 16.88% of mothers are in the poorest category, and 20.15% are in the poor at the present study. In order to improve the utilisation of MHC, health professionals need to reach and facilitate mothers who live in poverty.

The residential area was found as the determinant of institutionalised delivery and the use of PNC services. Mothers who settled in the urban area were more likely to give birth at healthcare facilities and unlikely to utilised PNC services. Regarding institutionalised delivery, those who resided in urban areas had the privilege of a broad range of choices and had better access to the facility that provides delivery care. Unlike the urban area, mothers who settled in the rural or countryside often face difficulties in finding and reaching healthcare facilities because their residence area was relatively least developed. ^19^^,^^20^ Apart from that, we discovered that mothers who settled in urban areas were unlikely to utilise PNC services. This might be attributed to mothers' misconceptions about insurance coverage. For instance, a previous qualitative study in Indonesia reported that some mothers thought national insurance cards could be used only for particular health care providers, such as the village midwife, and also they did not think it can be used for PNC. ^21^ This finding is not exclusive to Indonesia, as studies from other countries also demonstrated similar findings. ^14^^,^^15^^,^^22^ Thus, providing equal services for mothers who settle in urban and rural areas is warranted.

In favour of access to the healthcare facility, mothers who thought there was no problem tend to have adequate ANC visits and used the PNC service. Mothers may perceive numerous aspects could be causes of accessibility problems to a healthcare facility. However, few elements that often become problems are transportation, distance, medication, and treatment. More importantly, transportation and distance are classical problems in Indonesia, where infrastructures developed disproportionately across the country. ^23^ Such issues significantly hinder mothers from initiating ANC and receiving PNC. ^24^^,^^25^ Although approximately 89% of mothers thought there was no problem accessing healthcare facilities in the present study, we thought this remains important because mothers do not often speak up about this matter to the local health authority. Thus, more effort from the health professional to explore the unseen problem in accessing healthcare facilities among mothers is necessary.

With regard to the number of children, we found it was a determinant of ANC and PNC among Indonesian mothers. Compared to mothers who do not have children, those who had 1 to 3 children were more likely to have adequate ANC visits and PNC use, which was aligned with past works. ^13^^,^^14^ This was thought that as mothers became more experienced in motherhood and had appropriate knowledge from previous maternal experiences, they were encouraged to use those services. ^26^^,^^27^ In other words, those who were in their first-time pregnancy or delivery were at risk for underutilised ANC and PNC. We recognised that this finding might become an opportunity for health professionals to empower experienced mothers to encourage first-time mothers to use MHC correctly. However, experimental studies are required to prove this idea.

Husband’s educational level revealed as the determinant factor for institutionalised delivery and the use of PNC after delivery. Those with husbands who received secondary education have a greater likelihood to delivery at the healthcare facility and received PNC services. Husband’s education level may be linked with better health awareness that may make the family aware and utilise healthcare services better. ^28^ Also, since the Indonesian culture adopted the patriarchy concept, the husband plays a major role in family decision-making. Subsequently, a husband with better education may lead the family to utilise MHC properly.

The present study demonstrated that husbands’ work statuses determine the utilisation of ANC service. Husbands’ occupational status is thought to be linked with family income, which may later influence mothers’ ability to access ANC service. ^29^^,^^30^ It seems plausible because ANC recommendation in Indonesia is at least four times visits. However, for those financially and geographically disadvantaged may be discouraged from visiting the healthcare facility to receive ANC service. Most probably, the husbands who are working are relatively more economically prepared to face the mother’s needs during the pregnancy period.

This study identified the mother’s educational level associated with institutionalised delivery that was also supported by other studies. ^14^^,^^20^ Mothers who hold secondary education are prone to having delivery at the healthcare facility. This result is plausible since education is a marker for various factors that affect health-seeking behaviours. Compared to those with lower education levels, women with higher levels of education possess the level of health literacy required to make the right choices about their health and are better placed to overcome the cultural barriers to maternal health care use. ^31^^,^^32^ Moreover, low education can create a social distance between pregnant women and service providers, leading to poor quality client-provider interactions and discouraging services among women with low education. ^33^

A mother’s occupational status also has a role in determining PNC utilisation. This result aligns with previous studies, where the risk for underutilised PNC among working mothers was lower than a mothers who were not employed. ^12^^,^^14^ The possible explanation is that working mothers can control their earnings, preventing them from suffering from financial hardship and increasing their independence to seek healthcare services. ^34^ However, it should be noted that attending PNC requires mothers to spare their time from work which could cost working mothers loss of their incomes. Thus, providing flexible service hours and availability of maternity leave would help working mothers removing their barriers in accessing health services.

The data of this population-based study were collected by government bodies that have authority in maternal health surveys, namely the Central of Statistics Agency and the National Population and Family Planning Board, Indonesia. Also, study’s findings are generated from a large sample size that tends to provide high statistical power. However, despite the strength of this study, the present study also has some limitations that should be considered. To begin with, the cross-sectional nature adopted in this study cannot be over-interpreted for implying causality. In addition, the answer given by mothers were self-reported and based on their recall preceding survey that has the potential to result in bias. Furthermore, this study used data from a nationally representative sample; hence, this finding may be generalised to Indonesian mothers only.

## Conclusion

This population-based study revealed that the mother’s age and household wealth index were the typical determinants for each maternal healthcare service utilisation. Additionally, there were variabilities of determinant factors in each service. Provides a programme tailored with these determinant factors may ensure MHC can be accessed and adequately utilised by mothers. Besides, the programme may look into the health care services policy in reaching the underprivileged group and poor accessibility, like mobile health services and peer influence. Furthermore, engaging the father in the issue of MHC should be redesigned and rebranding especially for the young family.

## Data availability

Data used in this study are from the standard DHS VII recode dataset of the Indonesian 2017 Demographic and Health Survey (DHS) available from the Demographic and Health Survey (DHS) website. Access to the dataset requires registration and is granted only for legitimate research purposes. A guide for how to apply for dataset access is available at: https://dhsprogram.com/data/Access-Instructions.cfm. Other researchers will be able to access the data set in the same way as the authors and the authors do not have special access rights that others do not have.
