# Supplementary material for: Determinants of maternal healthcare service utilisation among Indonesian mothers: A population-based study
Source: F1000Res. 2022 Apr 21;10:1124. Originally published 2021 Nov 8. [Version 2] doi: 10.12688/f1000research.73847.2 (PMC9086521; doi:10.12688/f1000research.73847.2)
Supplement: Peer review history

# Peer review: Reviewer response for version 2

**DOI:** 10.5256/f1000research.132419.r135414

**Published:** 2022-05-09

**Authors:** Danish Ahmad

**Affiliations:** 1 Health Research Institute, Faculty of Health, University of Canberra, Canberra, ACT, Australia; 2 Public Health Foundation of India, Gurugram, India; 3 Australian National University, Canberra, ACT, Australia

The revised paper addresses my comments. While further improvements in the presentation of results and language can be made, these are mostly non-essential. I recommend the indexing of the revised version.

Is the work clearly and accurately presented and does it cite the current literature?

No

If applicable, is the statistical analysis and its interpretation appropriate?

Yes

Are all the source data underlying the results available to ensure full reproducibility?

Yes

Is the study design appropriate and is the work technically sound?

No

Are the conclusions drawn adequately supported by the results?

Yes

Are sufficient details of methods and analysis provided to allow replication by others?

Yes

Reviewer Expertise:

Global health, maternal health, health system strengthening.

I confirm that I have read this submission and believe that I have an appropriate level of expertise to confirm that it is of an acceptable scientific standard.

# Peer review: Reviewer response for version 1

**DOI:** 10.5256/f1000research.77532.r121898

**Published:** 2022-02-28

**Authors:** Danish Ahmad

**Affiliations:** 1 Health Research Institute, Faculty of Health, University of Canberra, Canberra, ACT, Australia; 2 Public Health Foundation of India, Gurugram, India; 3 Australian National University, Canberra, ACT, Australia

The research findings provide guidance to improve maternal health care seeking in Indonesia. The use of a nationally validated dataset allows findings to be generalisable for the country. The paper's contribution needs to be set against global maternal health research which has conclusively established the determinants of maternal health.

 In the current format, the paper's readability is affected by inconsistencies in language - this can however be remedied.

 Indonesia as an archipelago provides an opportunity to review maternal health determinants across different provinces and regions in Indonesia. The paper's focus on maternal health determinants needs further clarity in places.

 In table 2, the access to health services as being categorised as 'no problem' wasn't immediately clear. Please consider explaining how the variable was used.

 The discussion section is rather wordy and repetitive. The authors may like to use a framework such as the social determinants of health or Andersons health care seeking model to present findings. Another option is to present across individual, household and community/health system headings. The authors may also like to consider the program implications arising from this research.

Is the work clearly and accurately presented and does it cite the current literature?

No

If applicable, is the statistical analysis and its interpretation appropriate?

Yes

Are all the source data underlying the results available to ensure full reproducibility?

Yes

Is the study design appropriate and is the work technically sound?

No

Are the conclusions drawn adequately supported by the results?

Yes

Are sufficient details of methods and analysis provided to allow replication by others?

Yes

Reviewer Expertise:

Global health, maternal health, health system strengthening.

I confirm that I have read this submission and believe that I have an appropriate level of expertise to confirm that it is of an acceptable scientific standard, however I have significant reservations, as outlined above.

# Response

**Published:** 2022-04-16

**Authors:** Ferry Efendi

**Affiliations:** Universitas Airlangga, Indonesia

1. Many thanks for the reviewer’s suggestion about "The paper's contribution needs to be set against global maternal health research". We have added this information. Please see at the end of the first paragraph in the discussion section.
2. Many thanks for your on-point comment regarding the readability. The manuscript has been proofed by an English editor in the current version.
3. Thanks a lot for this query, in this study, we focused on rural and urban areas which comprised of 12033 residential areas in Indonesia.
4. We have added the concept definition of access to healthcare facilities.
5. Thank you very much for bringing this constructive feedback to our study. We have revised the discussion section and organized it based on the social determinant framework. Please see the discussion section.
6. We have revised the conclusion section.

# Peer review: Reviewer response for version 1

**DOI:** 10.5256/f1000research.77532.r120405

**Published:** 2022-02-23

**Authors:** Kusrini S. Kadar

**Affiliations:** 1 Faculty of Nursing, Hasanuddin University, Makassar, Indonesia

Overall this is an interesting study and it provides new insight to improve the healthcare facilities and services for pregnant mothers. 

 However, the authors need to explain more about what kind of services that the mothers received in the ANC, INC and PNC, especially at the PNC as it is interesting why mothers who live in urban area use the PNC services less. There is no clear explanation as the authors only compare this situation with other countries and due to insurance issues. In the conclusion, it seems that the authors give new ideas but not conclusions from the results; for example using mobile health services and peer influence are not the conclusions of the results. Perhaps this is more a recommendation than conclusion.

Is the work clearly and accurately presented and does it cite the current literature?

Yes

If applicable, is the statistical analysis and its interpretation appropriate?

I cannot comment. A qualified statistician is required.

Are all the source data underlying the results available to ensure full reproducibility?

Yes

Is the study design appropriate and is the work technically sound?

Yes

Are the conclusions drawn adequately supported by the results?

Partly

Are sufficient details of methods and analysis provided to allow replication by others?

Yes

Reviewer Expertise:

community health, family health, health education, health promotion

I confirm that I have read this submission and believe that I have an appropriate level of expertise to confirm that it is of an acceptable scientific standard.

# Response

**Published:** 2022-04-16

**Authors:** Ferry Efendi

**Affiliations:** Universitas Airlangga, Indonesia

1. We appreciate the reviewer’s positive feedback and recommendations. We added the explanations about ANC, INC, and PNC in the introduction to make sure the reader can understand these concepts earlier.  Please see the second paragraph.
2. Thank you for your feedback. Regarding “there is no clear explanation as to the authors only comparing this situation with other countries and due to insurance issues”, we have revised and added more explanation in the discussion section. Please see the second paragraph in the sub-section of intermediary determinants.
3. Thank you for your constructive feedback. We have revised our conclusion and made it more coherent with our findings.
